# Supplementary material for: Exploring the Usability of α-MSH-SM-Liposome as an Imaging Agent to Study Biodegradable Bone Implants In Vivo
Source: Int J Mol Sci. 2023 Jan 6;24(2):1103. doi: 10.3390/ijms24021103 (PMC9866773; doi:10.3390/ijms24021103)
Supplement: Supplementary file 1 [file ijms-24-01103-s001.zip › ijms-2101981-supplementary.pdf]

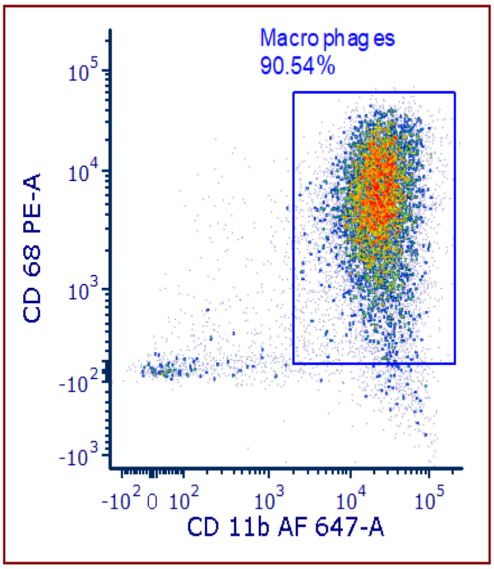

Figure S1. FACS analysis of BMDM purity of >90% (CD 68—P.E. for macrophages, CD 11b—Alexa flour 647 for hematopoietic cells).

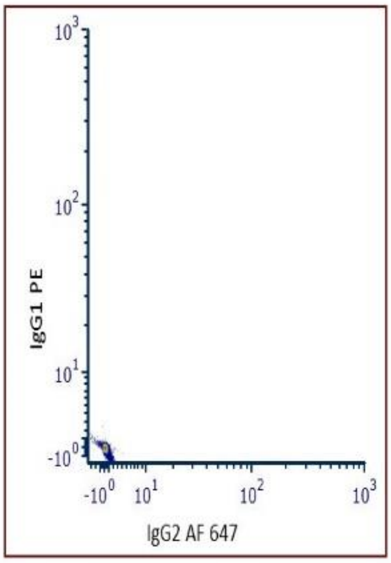

Figure S2. Isotype staining of BMDM with IgG1-PE and IgG2-AF 647.
